# Trends in COVID-19 Vaccine Administration and Effectiveness Through October 2021

**DOI:** 10.1001/jamanetworkopen.2022.5018

**Published:** 2022-03-31

**Authors:** Tyler N. A. Winkelman, Nayanjot K. Rai, Peter J. Bodurtha, Alanna M. Chamberlain, Malini DeSilva, Jessica Jeruzal, Steven G. Johnson, Anupam Kharbanda, Niall Klyn, Pamela J. Mink, Miriam Muscoplat, Stephen Waring, Yue Yu, Paul E. Drawz

**Affiliations:** 1 Division of General Internal Medicine, Department of Medicine, Hennepin Healthcare, Minneapolis, Minnesota; 2 Health, Homelessness, and Criminal Justice Lab, Hennepin Healthcare Research Institute, Minneapolis, Minnesota; 3 Division of Nephrology and Hypertension, University of Minnesota Medical School, Minneapolis; 4 Department of Quantitative Health Sciences, Mayo Clinic, Rochester, Minnesota; 5 HealthPartners Institute, Minneapolis, Minnesota; 6 Institute for Health Informatics, University of Minnesota, Minneapolis; 7 Department of Pediatric Emergency Medicine, Children’s Minnesota, Minneapolis; 8 Department of Information Services, Essentia Health, Duluth, Minnesota; 9 School of Communication, Northwestern University, Evanston, Illinois; 10 Health Economics Program, Health Policy Division, Minnesota Department of Health, Saint Paul; 11 Division of Infectious Disease, Epidemiology, Prevention, and Control, Minnesota Department of Health, Saint Paul; 12 Essentia Institute of Rural Health, Duluth, Minnesota

## Abstract

**Importance:**

COVID-19 vaccines are effective, but inequities in vaccine administration and waning immunity may limit vaccine effectiveness.

**Objectives:**

To report statewide trends in vaccine administration and vaccine effectiveness in Minnesota.

**Design, Setting, and Participants:**

This cohort study used COVID-19 vaccine data from the Minnesota Immunization Information Connection from October 25, 2020, through October 30, 2021 that were linked with electronic health record (EHR) data from health systems collaborating as part of the Minnesota EHR Consortium (MNEHRC). Participants included individuals who were seen at a participating health system in Minnesota.

**Exposures:**

Individuals were considered fully vaccinated in the second week after receipt of a second dose of a BNT162b2 or mRNA-1273 vaccine or a single dose of an Ad26.COV.2.S vaccine.

**Main Outcomes and Measures:**

A completed vaccination series and vaccine breakthrough, defined as either a positive SARS-CoV-2 polymerase chain reaction (PCR) test or a hospital admission the same week or within the 3 weeks following a positive SARS-CoV-2 PCR test. A test-negative design and incident rate ratio were used to evaluate COVID-19 vaccine effectiveness separately for the BNT162b2, mRNA-1273, and Ad26.COV.2.S vaccines. Rurality and social vulnerability index were assessed at the area level.

**Results:**

This study included 4 431 190 unique individuals at participating health systems, and 3 013 704 (68%) of the individuals were fully vaccinated. Vaccination rates were lowest among Minnesotans who identified as Hispanic (116 422 of 217 019 [54%]), multiracial (30 066 of 57 412 [52%]), American Indian or Alaska Native (22 190 of 41 437 [54%]), and Black or African American (158 860 of 326 595 [49%]) compared with Minnesotans who identified as Asian or Pacific Islander (159 999 of 210 994 [76%]) or White (2 402 928 of 3 391 747 [71%]). Among individuals aged 19 to 64 years, vaccination rates were lower in rural areas (196 479 of 308 047 [64%]) compared with urban areas (151 541 of 1 951 265 [77%]) and areas with high social vulnerability (544 433 of 774 952 [70%]) compared with areas with low social vulnerability (571 613 of 724 369 [79%]). In the 9 weeks ending October 30, 2021, vaccine effectiveness as assessed by a test-negative design was 33% (95% CI, 30%-37%) for Ad26.COV.2.S; 53% (95% CI, 52%-54%) for BNT162b2; and 66% (95% CI, 65%-67%) for mRNA-1273. For SARS-CoV-2–related hospitalizations, vaccine effectiveness in the 9 weeks ending October 30, 2021, was 78% (95% CI, 75%-81%) for Ad26.COV.2.S; 81% (95% CI, 79%-82%) for BNT162b2; and 81% (95% CI, 79%-82%) for mRNA-1273.

**Conclusions and Relevance:**

This cohort study of data from a Minnesota statewide consortium suggests disparities in vaccine administration and effectiveness. Vaccine effectiveness against infection was lower for Ad26.COV.2.S and BNT162b2 but was associated with protection against SARS-CoV-2–related hospitalizations despite the increased prevalence of the Delta variant in Minnesota.

## Key Points

**Question:**

Were there inequities in COVID-19 vaccine administration and vaccine effectiveness in September and October 2021 in Minnesota?

**Findings:**

In this observational study of 4 431 190 individuals from Minnesota, vaccination rates were lowest among Minnesotans who identified as Hispanic, multiracial, American Indian or Alaska Native, and Black or African American. Vaccine effectiveness against SARS-CoV-2–related hospitalization was 78% to 81% but lower in individuals aged 65 years and older and those with comorbidities.

**Meaning:**

These findings suggest that targeted interventions are needed to increase COVID-19 vaccination in populations with low vaccination rates and booster doses in populations at higher risk for SARS-CoV-2–related hospitalizations.

## Teaser

This cohort study reports statewide trends in vaccine administration and vaccine effectiveness in Minnesota.

## Introduction

Substantial inequities in COVID-19 vaccine administration exist among patient characteristics, including race, ethnicity, and geographic location. These inequities have contributed to persistent disparities in SARS-CoV-2 infection rates.^1,2,3^ Randomized clinical trials have demonstrated the efficacy of vaccines for preventing lab-confirmed and severe COVID-19 disease.^4,5,6^ The effectiveness of these vaccines was a component contributing to decreased SARS-CoV-2 infection rates in the summer of 2021.^7^ However, public health surveillance data in the United States have generally lacked the detail needed to make nuanced decisions about population-level vaccination programs and have a limited ability to merge vaccine and testing data. This information is critical to determine where additional vaccine-related policies and interventions are needed and to monitor vaccine effectiveness in near real-time as concerns increase about potential waning immunity and vaccine breakthrough cases.^8,9,10^

The US National Academy of Medicine recommends using electronic health record (EHR) data for disease monitoring and tracking, targeting medical services, and more effective and efficient research in routine medical care.^11,12^ The Minnesota Electronic Health Record Consortium (MNEHRC) includes 11 large Minnesota health systems that represent 77% of hospital admissions in the state.^13^ MNEHRC uses a distributed data network with linkage to COVID-19 vaccination data from the Minnesota Immunization Information Connection (MIIC). MNEHRC collaborated with the Minnesota Department of Health (MDH) on COVID-19 surveillance and monitoring of rates of COVID-19 vaccination for populations of interest.^14^ Linking vaccine and EHR data for public health surveillance also enabled statewide monitoring of and potential waning of vaccine effectiveness.

In this cohort study, we describe the current results of the MNEHRC vaccine monitoring as well as data through October 2021 about vaccine effectiveness across Minnesota. We used both a test-negative design and incident rate ratio (IRR) to evaluate vaccine effectiveness by manufacturer and time since the final dose of vaccination.

## Methods

This cohort study followed the Strengthening the Reporting of Observational Studies in Epidemiology (STROBE) reporting guideline. The study was reviewed by the institutional review board at each data-contributing site and was approved or deemed to be exempt as non-human patient research or a public health surveillance project. Informed consent was waived because EHR data were used, and the project was deemed to be for public health surveillance.

In the first quarter of 2020, individuals from several Minnesota health systems, statewide health care organizations, and MDH started to discuss opportunities for collaboration on research projects related to the epidemiology of chronic conditions like cardiovascular disease, hypertension, and substance use disorders. The COVID-19 pandemic provided a concrete use case for collaboration and led to the development of MNEHRC and its first project, a statewide EHR-based COVID-19 surveillance system with MDH.^15^ In January 2021, this collaboration expanded by partnering with MIIC to monitor the COVID-19 vaccine response.

### Electronic Health Record Data

MNEHRC includes 11 Minnesota health systems that contribute data: Allina Health, CentraCare, Children’s Minnesota, Essentia Health, HealthPartners, Hennepin Healthcare, M Health Fairview, Mayo Clinic, Minneapolis VA, Sanford Health, and North Memorial Health. Each of the 11 health care systems maintains a site-specific common data file with EHR data at the individual per week level. Individuals of all ages are included in these files for any week they had an event defined by any of the following: (1) collection of a SARS-CoV-2 polymerase chain reaction (PCR) or influenza test; (2) receipt of a COVID-19 vaccine; (3) a medical encounter that included an *International Statistical Classification of Diseases and Related Health Problems, Tenth Revision *(*ICD-10*) code that indicated possible SARS-CoV-2 infection (eg, influenza-like-illness, cough, shortness of breath, fever; a full list of included *ICD-10* codes is in the Supplement); or (4) experienced homelessness or incarceration, which were ascertained via linkage to state records. Individuals are included in a site’s file each week they have a qualifying event; there can be multiple rows per individual, but only 1 row per individual per week.

In addition to the event data, each row in the site-specific common data files contains sociodemographic characteristics, including age, sex, race, and ethnicity (Hispanic ethnicity, non-Hispanic race including American Indian or Alaska Native, Asian or Pacific Islander, Black or African American, multiracial, White), and language (English, Spanish, Somali, other non-English languages). Race and ethnicity were determined at the time of registration and are self-reported characteristics at most health systems. Comorbidities were based on the presence of 2 or more *ICD-10* codes from January 1, 2016, until the event date (Supplement). Neighborhood level characteristics were determined based on the patient’s most recent zip code at the time of vaccination or the most recent event for unvaccinated individuals. Socioeconomic status was defined using the US Centers for Disease Control and Prevention’s (CDC) social vulnerability index at the zip code level.^16^ Rurality was defined at the zip code tabulation area (ZCTA) level using 2010 rural-urban commuting area (RUCA) codes from the US Department of Agriculture’s Economic Research Service and percent of individuals living in rural areas from the US Census ACS.^17^ ZCTAs were classified as urban if classified as urban by RUCA and had less than 50% of the population living in a rural area; rural if classified as rural by RUCA and had 50% or more of the population living in a rural area; exurban if classified as urban by RUCA and had 50% or more of the population living in a rural area; and small urban if classified as rural by RUCA and had less than 50% of the population living in a rural area. Each health system also generated a hospitalization file with admission and discharge dates and a link to the site-specific common data file at the individual level.

### Immunization and Death Data

MIIC stores immunization records for individuals vaccinated in or living in Minnesota. All providers, except federal partners, such as the VA, are required to report COVID-19 doses administered to MIIC. Data provided by MIIC to MNEHRC included week of administration and vaccine manufacturer name (eg, Pfizer, Moderna, Janssen, etc). MIIC data are linked to MNEHRC data using a secure privacy-preserving record linkage (PPRL) process.^18^ Incoming data with hashed identification numbers from MIIC were matched to EHR data via health system-provided hashed identifiers. In this way, we were able to match over 90% of MIIC records to MNEHRC data without sharing identifying information. Patients with a COVID-19 vaccination record in MIIC who were not matched to an EHR record were excluded from the present analyses. In order to avoid reporting vaccinations more than once, all individuals are assigned a health system based on the presence of demographic data and the relative frequency of visits over the last year at each health system. We defined individuals as fully vaccinated 2 or more weeks after receiving a second dose of a BNT162b2 (Pfizer-BioNTech) or mRNA-1273 (Moderna) vaccine or a single dose of Ad26.COV.2.S (Janssen) vaccine. Individuals were considered unvaccinated if there was no matching MIIC record of a COVID-19 vaccination. Vaccine records for US Department of Veteran Affairs (VA) patients were obtained from the VA EHR. We matched EHR data with state death records using the same privacy-preserving record linkage.

### Data Aggregation

Using common, distributed analytic code, each participating site produced summary data on COVID-19 vaccinations, laboratory testing, and hospitalization, which was then aggregated. Population denominators were determined from the site-specific common data files at each health system after deduplication using only the most recent 3 years of data and excluding individuals who had died.

### Outcomes

A vaccine breakthrough case was defined as an individual with a positive SARS-CoV-2 PCR test at least 2 weeks after receipt of a final dose of a vaccine regardless of clinic setting or the presence of symptoms.^8^ Each individual was counted only once per week, even if multiple events occurred during a single week. If an individual had both a negative and positive test in a particular week, the positive test was counted. A SARS-CoV-2–related hospitalization was defined as a hospital admission for any reason the same week or in the 3 weeks following a positive SARS-CoV-2 PCR test.^8^

### Statistical Analysis

Cumulative vaccination rates were calculated based on the number of individuals vaccinated divided by the number of unique individuals at participating health systems. A test-negative design and IRRs were used to evaluate COVID-19 vaccine effectiveness.^7^ A test-negative design evaluates vaccine effectiveness by comparing the odds of a positive SARS-CoV-2 PCR test in the vaccinated population to the odds among the unvaccinated. Hospitalization rates were calculated based on person-years at risk assessed weekly. Individuals were considered to be either unvaccinated (no record of receipt of any COVID-19 vaccine) or fully vaccinated (at least 2 weeks after a final dose of BNT162b2, mRNA-1273, or Ad26.COV.2.S). Partially vaccinated individuals were excluded from vaccine effectiveness analyses. Individuals vaccinated during the study period would be considered unvaccinated before receiving their first vaccine and then vaccinated when they met criteria outlined above. Follow-up time for vaccinated individuals was censored upon receiving an additional or booster dose of a COVID-19 vaccine. Vaccine effectiveness analyses were limited to August 29 through October 30, 2021, to capture the most recent 2 months’ data. Vaccine effectiveness for SARS-CoV-2–related hospitalization was calculated with the formula vaccine effectiveness = (1 – IRR) × 100. CIs were calculated using unconditional maximum likelihood estimation. Patients with missing data elements were excluded from subgroup analyses for that variable. All statistical analyses were performed using R statistical software version 4.0.3 (R Foundation for Statistical Computing). Analyses were conducted between November 1, 2021, and February 2, 2022. Point estimates and 2-sided 95% CIs were calculated.

## Results

Vaccination data were reported from October 25, 2020, through October 30, 2021, from all MNEHRC health systems. Vaccination data includes trial participants seen at a participating health system. During this time, MNEHRC data included nearly all (90%) individuals vaccinated in Minnesota and 50% of all positive SARS-CoV-2 cases reported to MDH.

### Demographics of Vaccine Administration

**Figure 1.**
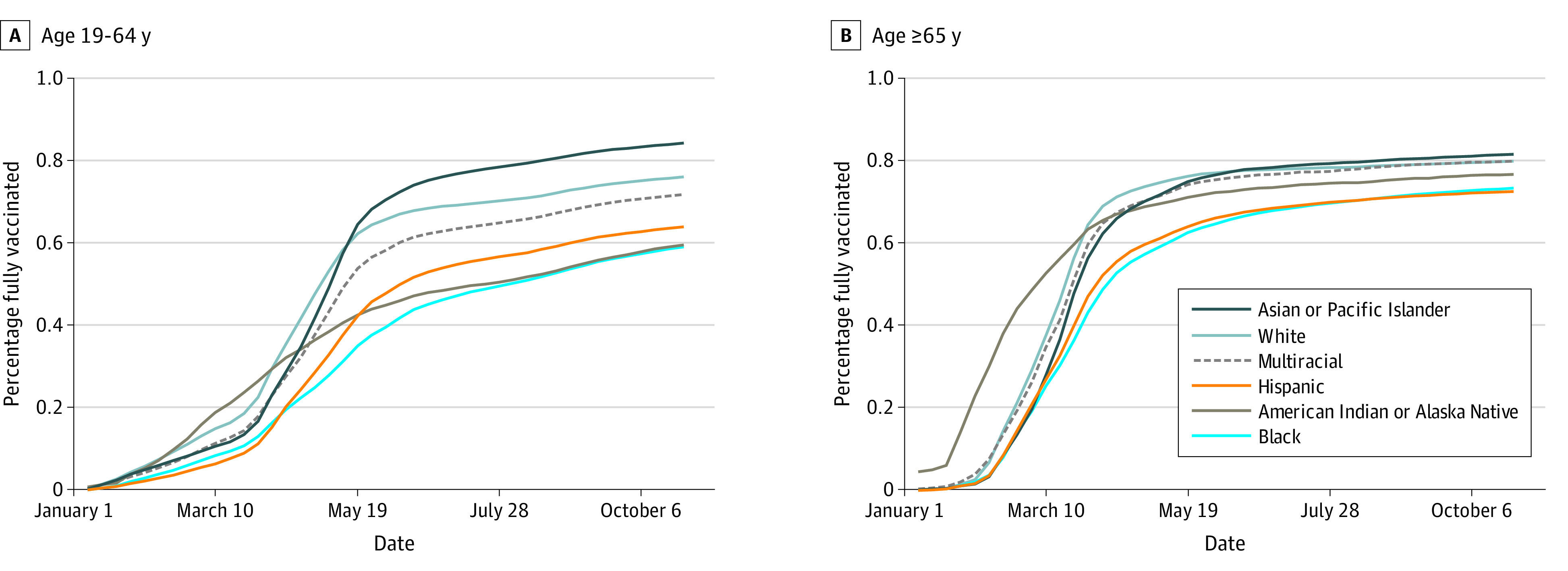
Cumulative Percentage Vaccinated by Racial and Ethnic Groups Among Those 19 to 64 Years of Age and 65 Years of Age and Older

There were 4 431 190 unique individuals in the site-specific data files at the participating health systems (Table). Approximately 3 million people received a full series of a COVID-19 vaccine. Vaccinations rates among Minnesotans who identified as White and Asian or Pacific Islander were higher compared with other race and ethnicity groups (Asian or Pacific Islander individuals: 159 999 of 210 994 [76%]; White individuals: 2 402 928 of 3 391 747 [71%]). Racial and ethnic disparities in vaccine administration were evident since early 2021, particularly in those 19 to 64 years of age (Figure 1). Vaccination rates in individuals who identified as American Indian or Alaskan Native were high in January and February 2021 relative to White individuals but have since declined. Cumulative vaccination rates were lowest among Hispanic individuals (116 422 of 217 019 [54%]), multiracial individuals (30 066 of 57 412 [52%]), American Indian or Alaskan Native individuals (22 190 of 41 437 [54%]), and Black or African American individuals (158 860 of 326 595 [49%]).

**Table.** Demographic Characteristics of Individuals Included in the Minnesota Electronic Health Record Consortium, Percent Vaccinated, and Postvaccine Percent Positive and Hospitalized, Through October 31, 2021

Group	Participants, No. (%)^b^	Participants, No. (%)^a^	
		Fully vaccinated^c^	Not fully vaccinated
Total	4 431 190	3 013 704 (68)	1 417 486 (32)
Age, y			
0-11	399 830 (9)	27 (<0.1)	399 803 (100)
12-18	370 202 (8)	246189 (67)	124 013 (33)
19-24	307 842 (7)	196 049 (64)	111 793 (36)
25-44	1 180 677 (27)	833 470 (71)	347 207 (29)
45-64	1 185 458 (27)	954 325 (81)	231 133 (19)
65-74	540 632 (12)	452 147 (84)	88 485 (16)
75 and older	442 481 (10)	328 576 (74)	113 905 (26)
Missing	4068 (<0.1)	2921 (72)	1147 (28)
Female	2 319 299 (52)	1 613 141 (70)	706 158 (30)
Male	2 110 649 (48)	1 400 759 (66)	709 890 (34)
American Indian or Alaska Native	41 437 (1)	22 190 (54)	19 247 (46)
Asian or Pacific Islander	210 994 (5)	159 999 (76)	50 995 (24)
Black	326 595 (7)	158 860 (49)	167 735 (51)
Hispanic	217 019 (5)	116 422 (54)	100 597 (46)
Multiracial	57 412 (1)	30 066 (52)	27 346 (48)
White	3 391 747 (77)	2 402 928 (71)	988 819 (29)
Other race or ethnicity^d^	185 321 (4)	123 665 (67)	61 656 (33)
Language			
English	4 057 767 (92)	2 766 438 (68)	1 291 329 (32)
Somali	46 997 (1)	19 909 (42)	27 088 (58)
Spanish	89 447 (2)	47 293 (53)	42 154 (47)
Used interpreter	146 809 (3)	76 247 (52)	70 562 (48)
Smoker	627 647 (14)	377 987 (60)	249 660 (40)
Hypertension	833 265 (19)	616 359 (74)	216 906 (26)
Diabetes	311 795 (7)	225 172 (72)	86 623 (28)
Heart disease	290 360 (7)	199 724 (69)	90 636 (31)
Cancer	235 248 (5)	176 306 (75)	58 942 (25)
Asthma	292 942 (7)	195 902 (67)	97 040 (33)
COPD	262 900 (6)	164 017 (62)	98 883 (38)
Chronic kidney disease	192 189 (4)	141 718 (74)	50 471 (26)
HIV	6760 (<0.1)	4926 (73)	1834 (27)
Substance use disorder	264 720 (6)	169 967 (64)	94 753 (36)
Urban	3 128 436 (71)	2 201 195 (70)	927 241 (30)
Exurban	241 230 (5)	153 192 (64)	88 038 (36)
Small urban	444 544 (10)	282 573 (64)	161 971 (36)
Rural	576 378 (13)	357 419 (62)	218 959 (38)
SVI quartile			
1	1 279 460 (29)	820 473 (64)	458 987 (36)
2	950 402 (21)	648 142 (68)	302 260 (32)
3	966 414 (22)	665 925 (69)	300 489 (31)
4	1 184 635 (27)	853 692 (72)	330 943 (28)

Abbreviations: COPD, chronic obstructive pulmonary disease; SVI social vulnerability index.

^a^ Percent of row.

^b^ Percent of column.

^c^ Number of individuals fully vaccinated (receipt of 2 doses of either BNT162b2 or mRNA-1273 vaccine or a single dose of Ad26.COV.2.S) in the Minnesota Electronic Health Record Consortium data.

^d^ Each health system did not report the specific race and ethnicity categories included in other.

Inequities by area level factors were also evident among those aged 19 to 64 years. Among this group, those living in urban areas had the highest vaccination rate. Vaccination rates were lower in rural areas (196 479 of 308 047 [64%]) compared with urban areas (151 541 of 1 951 265 [77%]) (eFigure 1 in the Supplement); rates were also lower in areas with high social vulnerability (544 433 of 774 952 [70%]) compared with lower social vulnerability (571 613 of 724 369 [79%]) (eFigure 2 in the Supplement). There was less difference in vaccination rates among those aged 65 years and older by rurality and social vulnerability index.

### Vaccine Effectiveness: SARS-CoV-2 Positivity

**Figure 2.**
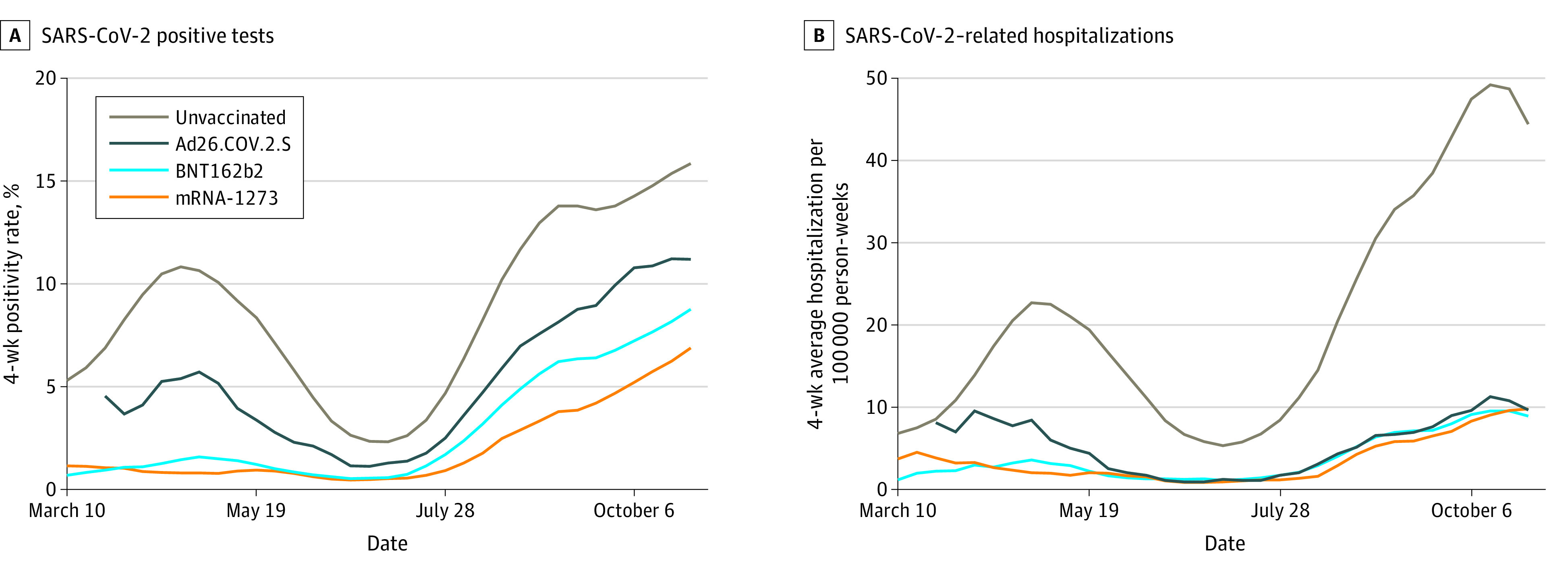
COVID-19 Breakthrough by Manufacturer as Assessed by SARS-CoV-2 Test Positivity and SARS-CoV-2–Related Hospitalizations

**Figure 3.**
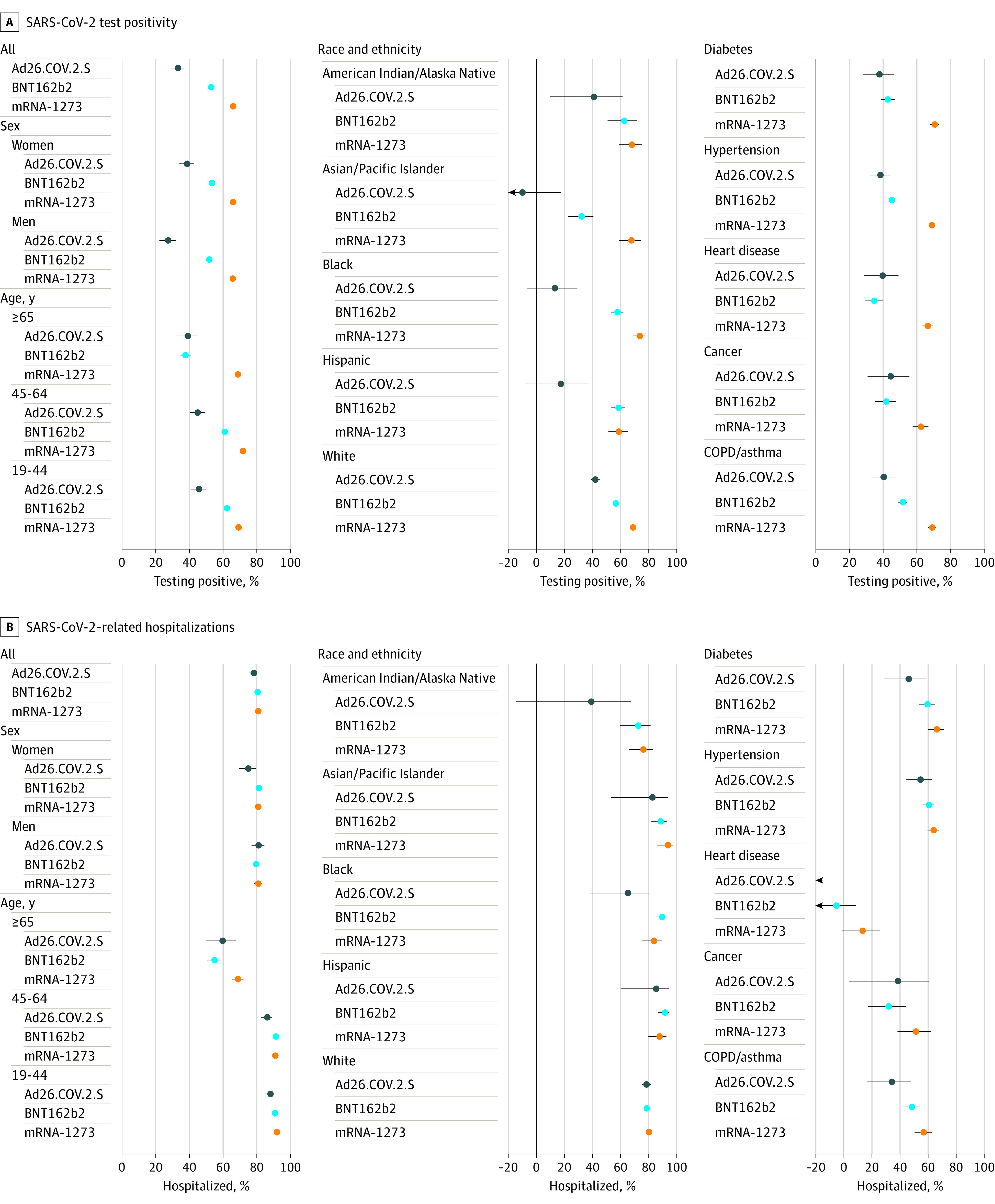
Vaccine Effectiveness Overall and by Subgroups as Assessed by SARS-CoV-2 Test Positivity and SARS-CoV-2–Related Hospitalizations, August 29 to October 30, 2021 COPD indicates chronic obstructive pulmonary disease.

Of more than 3 million individuals who were fully vaccinated in MNEHRC data, 252 502 were tested for SARS-CoV-2, of whom 17 566 (7%) had a positive PCR test between August 29 and October 30, 2021. Among 1.4 million unvaccinated individuals, 286 631 were tested, of whom 42 204 (15%) tested positive. In 2021, test positivity declined from a peak in March and April until mid-July when test positivity subsequently increased for both vaccinated and unvaccinated individuals. Across all weeks, the vaccinated population had lower test positivity than the unvaccinated population (Figure 2). Among the vaccinated, positivity rates were generally highest in those who had received the single-dose Ad26.COV.2.S vaccine followed by BNT162b2 with lower rates among those who received mRNA-1273 (Figure 2). In the 9 weeks ending October 30, 2021, vaccine effectiveness as assessed by a test-negative design was 33% (95% CI, 30-37) for Ad26.COV.2.S, 53% (95% CI, 52-54) for BNT162b2, and 66% (65-67) for mRNA-1273 (Figure 3; eTable 1 in the Supplement). Results varied by subgroup (Figure 3). Vaccine effectiveness was lower in individuals more than 26 weeks after vaccination compared with less than 26 weeks after vaccination for BNT162b2 and to a lesser extent mRNA-1273 (eFigure 3 in the Supplement).

### Vaccine Effectiveness: SARS-CoV-2–Related Hospitalizations

Of more than 3 million individuals fully vaccinated in MNEHRC data, 2042 had a SARS-CoV-2–related hospitalization between August 29 and October 30, 2021. As with test positivity, SARS-CoV-2–related hospitalizations were consistently higher in unvaccinated individuals and increased in vaccinated and unvaccinated individuals since mid-July 2021 (Figure 2). In July 2021, SARS-CoV-2–related hospitalization rates were 1 to 2 per 100 000 person-weeks in vaccinated individuals compared with 5 to 8 per 100 000 person-weeks in unvaccinated individuals. In October 2021, the rates were 9 per 100 000 person-weeks and 48 per 100 000 person-weeks in vaccinated and unvaccinated individuals, respectively. Vaccine effectiveness for SARS-CoV-2–related hospitalizations in the 9 weeks ending October 30, 2021, was 78% (95% CI, 75-81) for Ad26.COV.2.S, 81% (95% CI, 79-82) for BNT162b2, and 81% (95% CI, 79-82) for mRNA-1273 (Figure 3). Vaccine effectiveness was substantially lower in individuals aged 65 years and older and in those with diabetes, hypertension, heart disease, cancer, and chronic obstructive pulmonary disease or asthma (Figure 3). Vaccine effectiveness was lower in individuals more than 26 weeks after vaccination compared with less than 26 weeks after vaccination for BNT162b2 and mRNA-1273 (eFigure 3 in the Supplement).

## Discussion

The MNEHRC used a combination of EHR and statewide vaccine data to identify disparities in vaccine administration across a variety of different characteristics at the zip code level to provide more detailed information to decision-makers, other stakeholders, and the public. In terms of vaccine effectiveness, our data show that all 3 FDA-approved vaccines are associated with protection against SARS-CoV-2 and were highly effective at preventing hospitalization. SARS-CoV-2 positivity was higher among those who received an Ad26.COV.2.S vaccine followed by BNT162b2, with mRNA-1273 having the lowest positivity. Hospitalizations among individuals with a positive SARS-CoV-2 test were lower in those who received a COVID-19 vaccine than those who were unvaccinated. These findings add further evidence that vaccines are an effective tool for preventing SARS-CoV-2 and SARS-CoV-2–related hospitalizations, though differences exist between available vaccine manufacturers.

The 2-dose series of the mRNA-1273 or the BNT162b2 vaccines showed higher vaccine effectiveness for preventing SARS-CoV-2 positivity. Our findings are consistent with those of other recently published studies examining the comparative effectiveness of COVID-19 vaccines in preventing infections and associated hospitalizations. A recent study demonstrated higher vaccine effectiveness for mRNA-1273 (93%) or BNT162b2 (88%) compared with Ad26.COV.2.S (71%) in preventing hospitalizations.^10^ While our study showed similar vaccine effectiveness for preventing hospitalizations among the 3 vaccines, 2 studies from the CDC’s VISION network reported vaccine effectiveness for preventing hospitalizations of 91% to 95% for mRNA-1273, 80% to 87% for BNT162b2, and 60% to 68% for Ad26.COV.2.S.^7,19^ Waning vaccine effectiveness for BNT162b2 was demonstrated for infection but not hospitalization among individuals in the Kaiser Permanente Southern California system and individuals from Qatar.^8,9^ Our results are drawn from a larger population with a significantly greater number of hospitalizations among individuals vaccinated with BNT162b2, mRNA-1273, and Ad26.COV.2.S in the 9 weeks ending October 30, 2021, compared with the populations of the 3 CDC studies combined (BNT162b2: 1102 vs 426 hospitalizations; mRNA-1273: 734 vs 219 hospitalizations; and Ad26.COV.2.S: 206 vs 97 hospitalizations).^7,10,19^ Additionally, to our knowledge, this is the first US study with a geographic coverage that allowed for calculation of incident rates for SARS-CoV-2–related hospitalizations and the first evaluation of vaccine effectiveness using IRR.

The approach to vaccine monitoring and evaluation of vaccine effectiveness developed by the MNEHRC holds substantial promise in ongoing monitoring of COVID-19 vaccine administration and effectiveness. The distributed approach allowed for individual-level analyses and facilitated the rapid establishment and collaboration of the MNEHRC. The collaboration with MIIC enabled a 90% capture of COVID-19 vaccines administered in Minnesota, which allowed participating health systems to accurately determine which individuals were vaccinated regardless of where they received their vaccination. By relying on EHR data that do not require central aggregation, we were able to produce near real-time data across an entire state. Such an approach could be expanded regionally or nationally to ensure more timely and detailed data to support ongoing efforts to manage COVID-19 and monitor changes in vaccine effectiveness over time.

### Limitations

This study had limitations. First, the data summary represented by the MNEHRC analyses does not include complete statewide data. The vaccination data are nearly complete, however, the PCR testing data includes 50% of SARS-CoV-2 positive cases across Minnesota, and the health systems participating in the MNEHRC represent 77% of hospital admissions in the state. Second, our area level analyses are at the zip code level rather than the census tract because of the limited geocoding of addresses across the health systems. Third, hospitalizations around a SARS-CoV-2–positive test may not be related to the infection. However, 75% of SARS-CoV-2–related hospitalizations at a participating health system had a COVID–like illness *ICD-10* code.^19^ Finally, given the summary nature of the data from each site, estimates are unadjusted for individual characteristics. However, results are consistent across subgroups with lower vaccine effectiveness for SARS-CoV-2–related hospitalizations in those aged 65 years and older and those with diabetes, hypertension, heart disease, cancer, and chronic obstructive pulmonary disease or asthma.

## Conclusions

This cohort study of EHR data from 11 large health systems in Minnesota suggests vaccine administration and effectiveness disparities. Vaccine effectiveness against infection was lower for Ad26.COV.2.S and BNT162b2, but a high degree of protection from all 3 vaccines persisted against SARS-CoV-2–related hospitalizations through October 30, 2021, despite the increased prevalence of the Delta variant in Minnesota.

## Notes

Supplement. **eTable 1.** Vaccine Effectiveness for Medically Attended SARS-CoV-2 Infections by Manufacturer **eTable 2.** Vaccine Effectiveness for SARS-CoV-2–Related Hospitalizations by Manufacturer **eFigure 1.** Cumulative Percentage Vaccinated by Rurality Among Those 19 to 64 Years of Age and 65 Years of Age and Older **eFigure 2.** Cumulative Percentage Vaccinated by Social Vulnerability Index Among Those 19 to 64 Years of Age and 65 Years of Age and Older **eFigure 3.** Vaccine Effectiveness Overall and by Subgroups Based on Time Since Vaccination as Assessed by SARS-CoV-2 Test Positivity and SARS-CoV-2–Related Hospitalizations—August 29 to October 30, 2021
