# Supplementary material for: Trends in COVID-19 Vaccine Administration and Effectiveness Through October 2021
Source: JAMA Netw Open. 2022 Mar 31;5(3):e225018. doi: 10.1001/jamanetworkopen.2022.5018 (PMC8972031; doi:10.1001/jamanetworkopen.2022.5018)
Supplement: Supplement. — eTable 1. Vaccine Effectiveness for Medically Attended SARS-CoV-2 Infections by Manufacturer eTable 2. Vaccine Effectiveness for SARS-CoV-2–Related Hospitalizations by Manufacturer eFigure 1. Cumulative Percentage Vaccinated by Rurality Among Those 19 to 64 Years of Age and 65 Years of Age and Older eFigure 2. Cumulative Percentage Vaccinated by Social Vulnerability Index Among Those 19 to 64 Years of Age and 65 Years of Age and Older eFigure 3. Vaccine Effectiveness Overall and by Subgroups Based on Time Since Vaccination as Assessed by SARS-CoV-2 Test Positivity and SARS-CoV-2–Related Hospitalizations—August 29 to October 30, 2021 [file jamanetwopen-e225018-s001.pdf]

Supplemental Online Content

Winkelman TNA, Rai NK, Bodurtha PJ, et al. Trends in COVID-19 vaccine administration and effectiveness through October 2021. *JAMA Netw Open*. 2022;5(3):e225018. doi:10.1001/jamanetworkopen.2022.5018

eTable 1. Vaccine Effectiveness for Medically Attended SARS-CoV-2 Infections by Manufacturer
eTable 2. Vaccine Effectiveness for SARS-CoV-2–Related Hospitalizations by Manufacturer
eFigure 1. Cumulative Percentage Vaccinated by Rurality Among Those 19 to 64 Years of Age and 65 Years of Age and Older
eFigure 2. Cumulative Percentage Vaccinated by Social Vulnerability Index Among Those 19 to 64 Years of Age and 65 Years of Age and Older
eFigure 3. Vaccine Effectiveness Overall and by Subgroups Based on Time Since Vaccination as Assessed by SARS-CoV-2 Test Positivity and SARS-CoV-2–Related Hospitalizations—August 29 to October 30, 2021

This supplemental material has been provided by the authors to give readers additional information about their work.

© 2022 Winkelman TNA et al. *JAMA Network Open*.

eTable 1. Vaccine Effectiveness for Medically Attended SARS-CoV-2 Infections by Manufacturer								
		Vaccinated		Unvaccinated				
Group	Manufacturer	Tested	Positive	Tested	Positive	VE	VE 95% CI LL	VE 95% CI UL
All	JSN	18041	1859	286631	42204	33	30	37
All	PFR	140777	10538	286631	42204	53	52	54
All	MOD	93684	5169	286631	42204	66	65	67
Female	JSN	9634	887	150493	21381	39	34	43
Female	PFR	84727	6058	150493	21381	53	52	55
Female	MOD	55894	2973	150493	21381	66	65	67
Male	JSN	8407	972	136138	20823	28	22	32
Male	PFR	56050	4480	136138	20823	52	50	53
Male	MOD	37790	2196	136138	20823	66	64	67
Age 65 and up	JSN	4704	419	24526	3402	39	32	45
Age 65 and up	PFR	38521	3509	24526	3402	38	35	41
Age 65 and up	MOD	40722	1938	24526	3402	69	67	71
Age 45 to 64	JSN	6726	740	39520	7271	45	41	49
Age 45 to 64	PFR	39364	3170	39520	7271	61	59	63
Age 45 to 64	MOD	27653	1639	39520	7271	72	70	74
Age 19 to 44	JSN	6476	691	81695	14779	46	41	50
Age 19 to 44	PFR	43171	3305	81695	14779	62	61	64
Age 19 to 44	MOD	24938	1578	81695	14779	69	68	71
American Indian/Alaska Native MN	JSN	218	25	3605	650	41	10	62
American Indian/Alaska Native MN	PFR	792	60	3605	650	63	51	72
American Indian/Alaska Native MN	MOD	1057	69	3605	650	68	59	75
Asian/PI MN	JSN	534	55	8805	833	-10	-47	18
Asian/PI MN	PFR	4956	327	8805	833	32	23	41
Asian/PI MN	MOD	2243	73	8805	833	68	59	75

© 2022 Winkelman TNA et al. *JAMA Network Open*.

Black MN	JSN	1000	106	31406	3778	13	-6	29
		Vaccinated		Unvaccinated				
Group	Manufacturer	Tested	Positive	Tested	Positive	VE	VE 95% CI LL	VE 95% CI UL
Black MN	PFR	7257	395	31406	3778	58	53	62
Black MN	MOD	4343	151	31406	3778	74	69	78
Hispanic MN	JSN	526	63	19172	2711	17	-8	37
Hispanic MN	PFR	5199	331	19172	2711	59	54	63
Hispanic MN	MOD	2442	155	19172	2711	59	51	65
White MN	JSN	15126	1537	193725	31656	42	39	45
White MN	PFR	117485	9156	193725	31656	57	56	58
White MN	MOD	79952	4572	193725	31656	69	68	70
Diabetes	JSN	2141	224	11746	1859	38	28	46
Diabetes	PFR	15862	1539	11746	1859	43	39	47
Diabetes	MOD	13804	723	11746	1859	71	68	73
Hypertension	JSN	4971	518	27056	4301	38	32	44
Hypertension	PFR	37647	3533	27056	4301	45	43	48
Hypertension	MOD	32329	1794	27056	4301	69	67	71
Heart Disease	JSN	2256	166	10968	1276	40	29	49
Heart Disease	PFR	18499	1463	10968	1276	35	29	40
Heart Disease	MOD	17641	745	10968	1276	67	63	70
Cancer	JSN	1415	93	7206	811	45	31	56
Cancer	PFR	11014	757	7206	811	42	35	48
Cancer	MOD	8912	405	7206	811	62	58	67
COPD/Asthma	JSN	3857	337	33382	4610	40	33	47
COPD/Asthma	PFR	27916	2004	33382	4610	52	49	54
COPD/Asthma	MOD	20019	941	33382	4610	69	67	71
Note: MOD, Moderna; PFR, Pfizer; JSN, Janssen; VE, vaccine effectiveness.								

© 2022 Winkelman TNA et al. *JAMA Network Open*.

eTable 2. Vaccine Effectiveness for SARS-CoV-2–Related Hospitalizations by Manufacturer								
		Vaccinated		Unvaccinated				
Group	Manufacturer	Person weeks at risk	Hospitalizations	Person weeks at risk	Hospitalizations	VE	VE 95% CI LL	VE 95% CI UL
All	JSN	2224734	206	12847374	5485	78	75	81
All	PFR	13290171	1102	12847374	5485	81	79	82
All	MOD	8992791	734	12847374	5485	81	79	82
Female	JSN	1002927	105	6502524	2725	75	70	79
Female	PFR	7135878	558	6502524	2725	81	80	83
Female	MOD	4921100	395	6502524	2725	81	79	83
Male	JSN	1222373	101	6333672	2760	81	77	84
Male	PFR	6155906	544	6333672	2760	80	78	82
Male	MOD	4072822	339	6333672	2760	81	79	83
Age 65 and up	JSN	391972	87	2060310	1133	60	50	68
Age 65 and up	PFR	2791395	692	2060310	1133	55	50	59
Age 65 and up	MOD	2647700	454	2060310	1133	69	65	72
Age 45 to 64	JSN	837623	74	2169771	1398	86	83	89
Age 45 to 64	PFR	4054318	225	2169771	1398	91	90	93
Age 45 to 64	MOD	3107061	180	2169771	1398	91	89	92
Age 19 to 44	JSN	954696	44	4051770	1583	88	84	91
Age 19 to 44	PFR	4491210	161	4051770	1583	91	89	92
Age 19 to 44	MOD	3163822	99	4051770	1583	92	90	93
American Indian/Alaska Native MN	JSN	9936	<11	163485	270	40	-15	70
American Indian/Alaska Native MN	PFR	61613	28	163485	270	72	59	81
American Indian/Alaska Native MN	MOD	88431	35	163485	270	76	66	83
Asian/PI MN	JSN	98919	<11	428072	100	85	55	95

© 2022 Winkelman TNA et al. *JAMA Network Open*.

Asian/PI MN	PFR	824339	22	428072	100	89	82	93
Asian/PI MN	MOD	411412	<11	428072	100	95	85	95
Black MN	JSN	103499	12	1447221	483	65	38	80
Black MN	PFR	752624	26	1447221	483	90	85	93
Black MN	MOD	440830	24	1447221	483	84	75	89
Hispanic MN	JSN	66553	<11	889956	363	85	60	95
Hispanic MN	PFR	579412	20	889956	363	92	87	95
Hispanic MN	MOD	318371	16	889956	363	88	80	93
White MN	JSN	1797475	172	9190345	4082	78	75	82
White MN	PFR	10397863	992	9190345	4082	79	77	80
White MN	MOD	7236283	641	9190345	4082	80	78	82
Diabetes	JSN	129471	52	857796	639	46	28	59
Diabetes	PFR	900965	272	857796	639	59	53	65
Diabetes	MOD	718042	181	857796	639	66	60	71
Hypertension	JSN	369912	98	2117451	1233	55	44	63
Hypertension	PFR	2439743	558	2117451	1233	61	57	64
Hypertension	MOD	1948911	410	2117451	1233	64	60	68
Heart Disease	JSN	92353	62	1036670	463	-50	-96	-15
Heart Disease	PFR	732592	345	1036670	463	-5	-21	8
Heart Disease	MOD	638186	247	1036670	463	13	-1	26
Cancer	JSN	91023	21	626852	235	38	4	61
Cancer	PFR	669191	171	626852	235	32	17	44
Cancer	MOD	510129	93	626852	235	51	38	62
COPD/Asthma	JSN	226555	75	1992756	1002	34	17	48
COPD/Asthma	PFR	1533243	398	1992756	1002	48	42	54
COPD/Asthma	MOD	1062205	230	1992756	1002	57	50	63
Note: MOD, Moderna; PFR, Pfizer; JSN, Janssen; VE, vaccine effectiveness.								

© 2022 Winkelman TNA et al. *JAMA Network Open*.

eFigure 1. Cumulative Percentage Vaccinated by Rurality Among Those 19 to 64 Years of Age and 65 Years of Age and Older

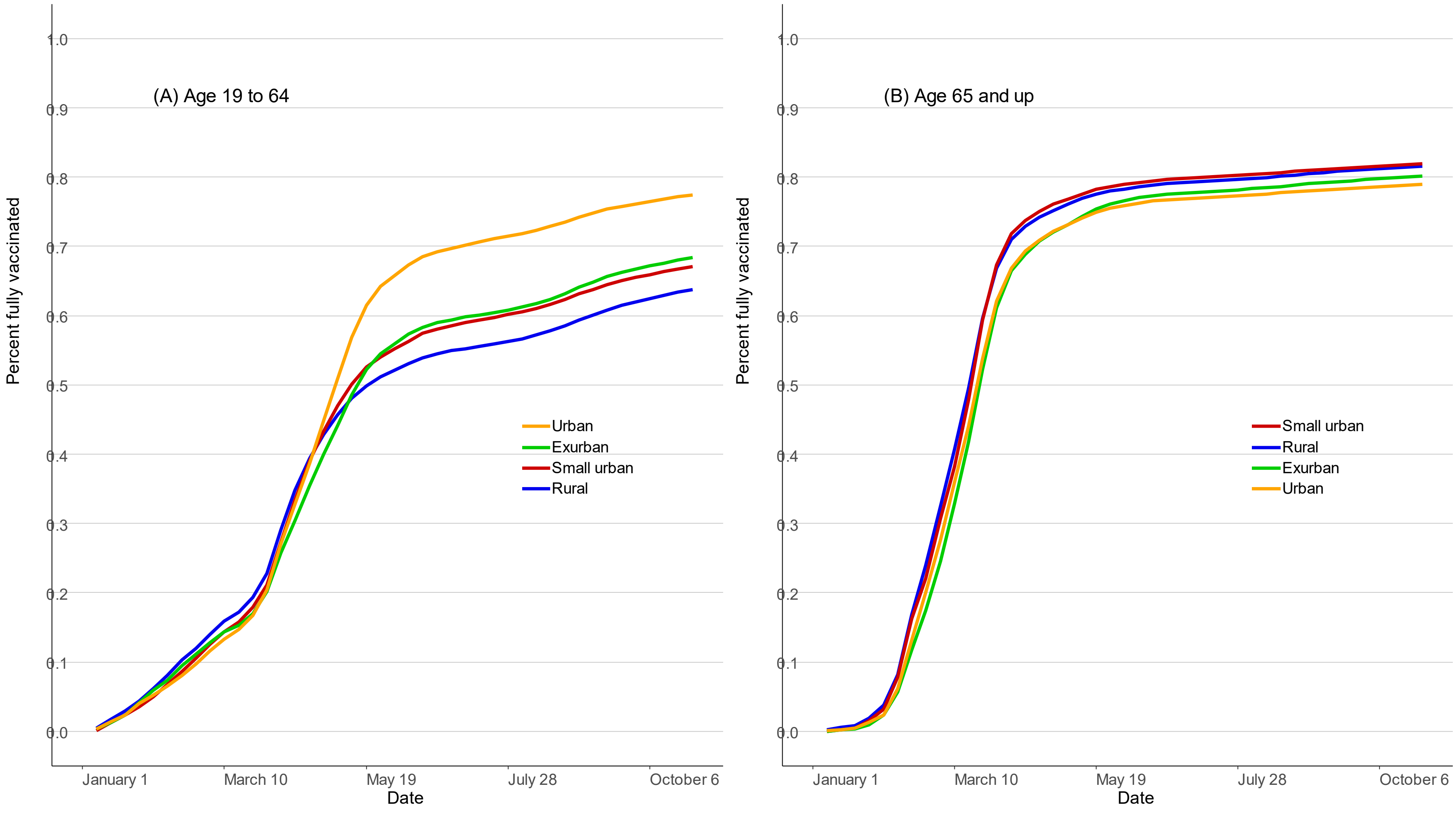

© 2022 Winkelman TNA et al. *JAMA Network Open*.

eFigure 2. Cumulative Percentage Vaccinated by Social Vulnerability Index Among Those 19 to 64 Years of Age and 65 Years of Age and Older

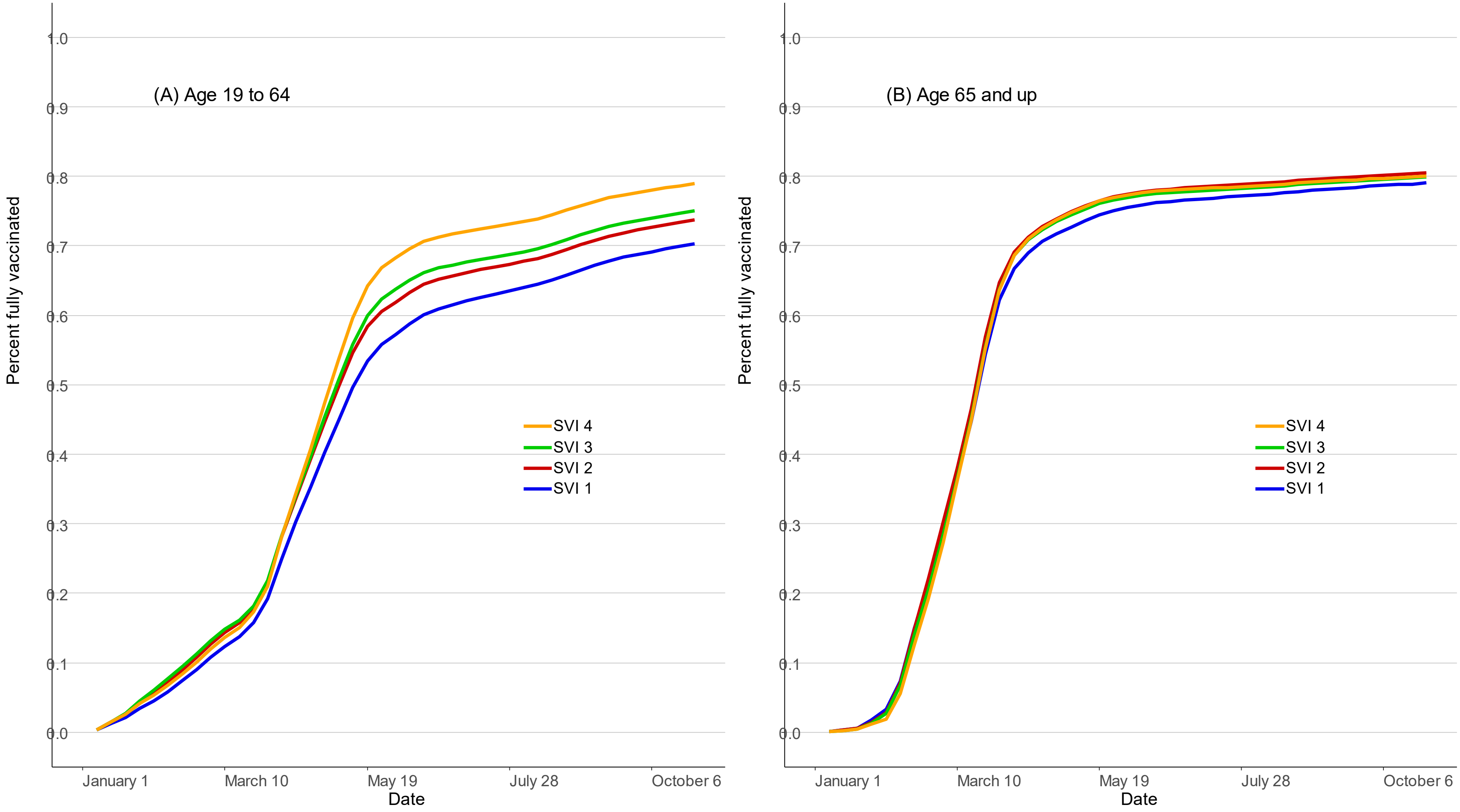

© 2022 Winkelman TNA et al. *JAMA Network Open*.

eFigure 3. Vaccine Effectiveness Overall and by Subgroups Based on Time Since Vaccination as Assessed by SARS-CoV-2 Test Positivity and SARS-CoV-2–Related Hospitalizations—August 29 to October 30, 2021

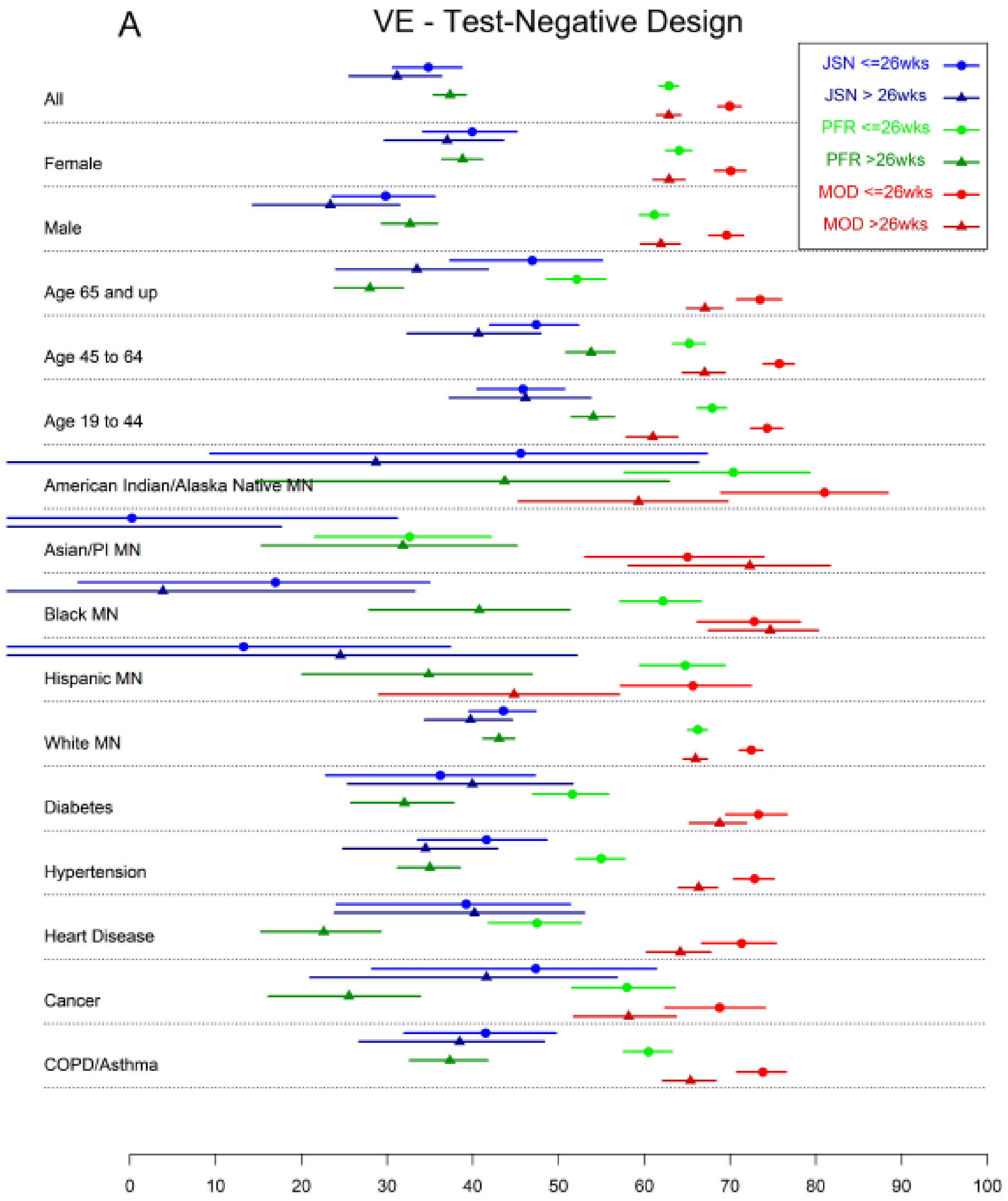

Note. Top (lighter color) line for each manufacturer is for the period 2 to 26 weeks after vaccination, bottom (darker) line is for the period more than 26 weeks after vaccination.

© 2022 Winkelman TNA et al. *JAMA Network Open*.

eFigure 3 continued. Vaccine Effectiveness Overall and by Subgroups Based on Time Since Vaccination as Assessed by SARS-CoV-2 Test Positivity and SARS-CoV-2–Related Hospitalizations—August 29 to October 30, 2021

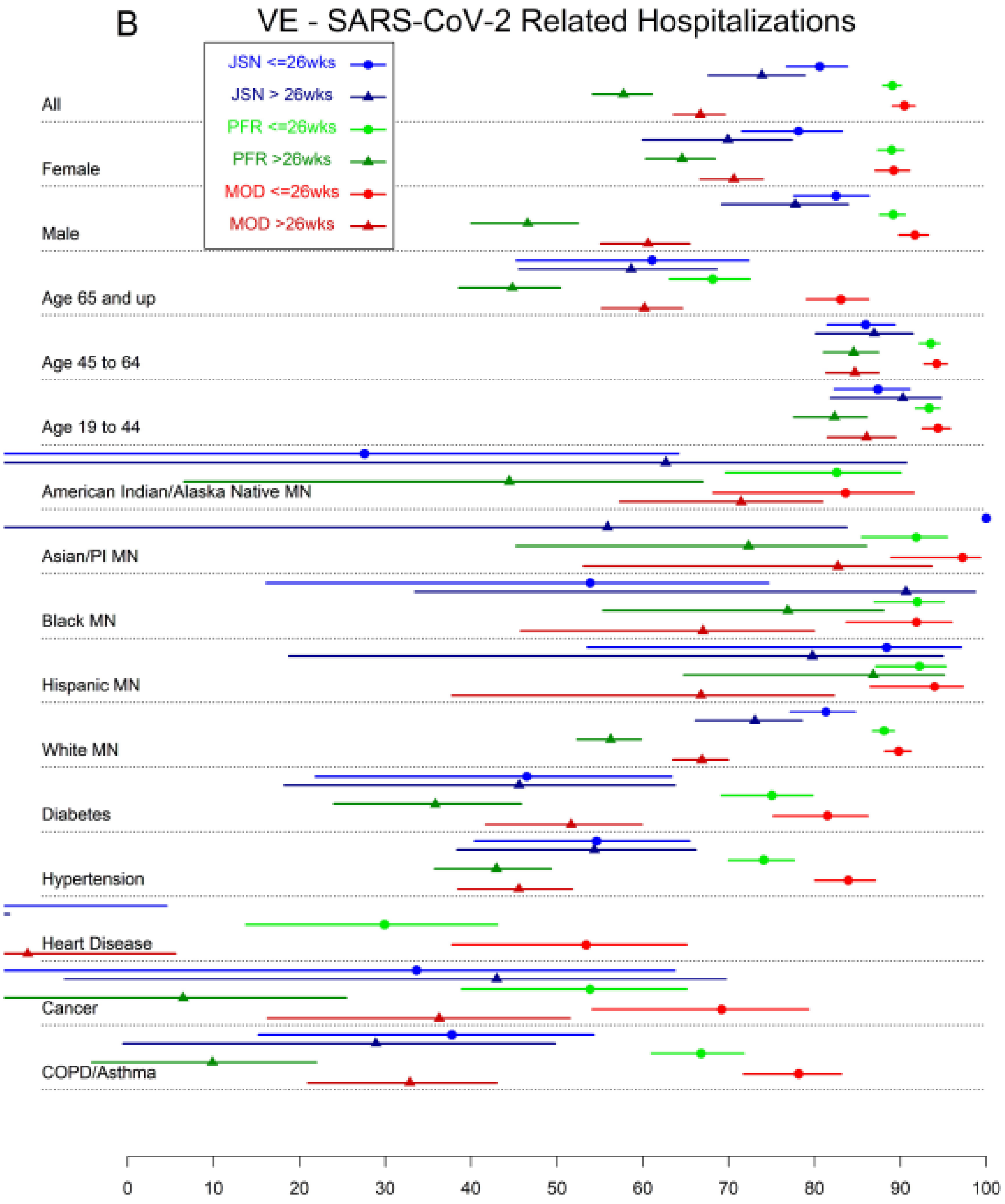

Note. Top (lighter color) line for each manufacturer is for the period 2 to 26 weeks after vaccination, bottom (darker) line is for the period more than 26 weeks after vaccination

© 2022 Winkelman TNA et al. *JAMA Network Open*.
